# Accumulation of Cerebrospinal Fluid, Ventricular Enlargement, and Cerebral Folate Metabolic Errors Unify a Diverse Group of Neuropsychiatric Conditions Affecting Adult Neocortical Functions

**DOI:** 10.3390/ijms251810205

**Published:** 2024-09-23

**Authors:** Lena Ikeda, Adrià Vilaseca Capel, Dhruti Doddaballapur, Jaleel Miyan

**Affiliations:** Division of Neuroscience, Faculty of Biology, Medicine & Health, School of Biological Science, The University of Manchester, 3.540 Stopford Building, Oxford Road, Manchester M13 9PT, UK; lena.ikeda@postgrad.manchester.ac.uk (L.I.); avilasca9@alumnes.ub.edu (A.V.C.); dhruti.doddaballapur@manchester.ac.uk (D.D.)

**Keywords:** cerebrospinal fluid, cerebral folate, FOLR1, ALDH1L1, FDH, neurological conditions

## Abstract

Cerebrospinal fluid (CSF) is a fluid critical to brain development, function, and health. It is actively secreted by the choroid plexus, and it emanates from brain tissue due to osmolar exchange and the constant contribution of brain metabolism and astroglial fluid output to interstitial fluid into the ventricles of the brain. CSF acts as a growth medium for the developing cerebral cortex and a source of nutrients and signalling throughout life. Together with perivascular glymphatic and interstitial fluid movement through the brain and into CSF, it also acts to remove toxins and maintain metabolic balance. In this study, we focused on cerebral folate status, measuring CSF concentrations of folate receptor alpha (FOLR1); aldehyde dehydrogenase 1L1, also known as 10-formyl tetrahydrofolate dehydrogenase (ALDH1L1 and FDH); and total folate. These demonstrate the transport of folate from blood across the blood–CSF barrier and into CSF (FOLR1 + folate), and the transport of folate through the primary FDH pathway from CSF into brain FDH + ve astrocytes. Based on our hypothesis that CSF flow, drainage issues, or osmotic forces, resulting in fluid accumulation, would have an associated cerebral folate imbalance, we investigated folate status in CSF from neurological conditions that have a severity association with enlarged ventricles. We found that all the conditions we examined had a folate imbalance, but these folate imbalances were not all the same. Given that folate is essential for key cellular processes, including DNA/RNA synthesis, methylation, nitric oxide, and neurotransmitter synthesis, we conclude that ageing or some form of trauma in life can lead to CSF accumulation and ventricular enlargement and result in a specific folate imbalance/deficiency associated with the specific neurological condition. We believe that addressing cerebral folate imbalance may therefore alleviate many of the underlying deficits and symptoms in these conditions.

## 1. Introduction

Cerebral neuropsychiatric conditions are those that affect higher cortical functions and include developmental conditions, such as autism, ADHD, epilepsy, and childhood versions of Schizophrenia and bipolar, as well as later/adult-onset conditions, also including epilepsy, Schizophrenia, bipolar, dementia, Parkinson’s, and Alzheimer’s. Although these conditions are many and varied in regard to pathology, functional effects, and outcomes, there are common elements when examined in terms of anatomical aetiology, for example. Remarkably, the findings of cerebrospinal fluid (CSF) accumulation, ventricular enlargement, and hydrocephalus, associated with severity of symptoms, are found in many conditions affecting the cerebral cortex. These include, but are not limited to, psychosis [1,2]; Schizophrenia [3]; bipolar disorder [4]; autism, in which CSF also accumulates in the subarachnoid space outside the ventricles as external hydrocephalus [5,6,7,8,9]; Parkinson’s disease [10,11], including consequences of treatment with L-DOPA [12]; epilepsy, including specifically related to use of the anti-epileptic drug valproate [13]; hypo-myelination [14]; and multiple sclerosis [15,16,17,18]. Disease severity has also been associated with increased ventricular enlargement in Alzheimer’s disease [19,20,21,22,23,24] and is a defining characteristic of normal-pressure hydrocephalus (NPH) [25], a condition with dementia, gait infirmity, and incontinence [26,27]. These observations indicate the potential operation of a common mechanism involving a CSF drainage insufficiency, change in CSF output, and/or osmotic changes linked to increased CSF macromolecule levels, leading to ventricular enlargement, along with physical and metabolic consequences on the cerebral cortex [28,29,30,31].

The choroid plexus is the recognised major source of CSF and actively secretes CSF into the ventricles of the adult brain, with production reportedly affected by flow or drainage obstructions [31]. Although secretion alone may be at a sufficient rate to expand the ventricles, this would also be affected by macromolecular concentration changes and resulting osmotic forces [28,29,30]. This would seem to indicate a critical role for CSF in cerebral cortical health and function, and this is receiving increasing research activity. As these conditions and observations present a spectrum of CSF accumulation, we hypothesise that a similar spectrum of cerebral folate metabolic issues will also be associated with these conditions, as we have found in neonatal and infant congenital hydrocephalus and, more recently, in Alzheimer’s disease [32,33,34,35,36]. The range of fluid-drainage obstruction and fluid accumulation is associated with different functional effects and outcomes in the conditions listed above, which may also be associated with specific cerebral folate issues. Moreover, as folate metabolism is key to many associated metabolic processes, the diverse effects and conditions that could result from this issue are equally many [33]. Studies already exist linking some of these conditions, as well as the underlying pathophysiology, to cerebral folate metabolic errors, thus supporting such a hypothesis [14,37,38,39,40,41,42,43,44,45,46,47,48,49].

The vital role of CSF as a growth medium in development and as a source of nutrients and metabolites for the function of the cerebral cortex has been discussed in the recent literature [50,51,52,53,54,55], highlighting the importance of CSF flow through the ventricles and subarachnoid spaces and drainage from the head to ensure the optimal development and function of the cortex [51,56,57]. In addition to the main CSF pathway through the ventricular system and subarachnoid spaces, glymphatic pathways involve perivascular CSF and interstitial fluid interactions. Mediated through astroglial water and ion transport to produce interstitial fluid movement/convection, the glymphatic fluid passes along perivascular pathways from CSF and back into CSF, as well as passing into and through the dura and into the dural sinuses and lymphatics. A major drainage pathway is the cribriform plate under the olfactory lobe of the brain. These glymphatic pathways have received focussed attention since interstitial fluid movement is essential for the removal of toxins, including amyloid and tau, from the brain parenchyma [58]. Where glymphatic function fails, or is not optimal, which is the case in multiple neurological conditions, including neurodegenerative conditions and brain trauma, and in disorders of sleep [59,60,61,62,63,64,65,66,67,68,69,70,71,72,73,74,75,76,77,78,79,80,81,82,83,84,85,86,87,88,89,90,91,92,93], interstitial movement of waste and toxins to the CSF is compromised. This may also occur due to macromolecules building up in the CSF, due to any pathology, and causing an osmotic pressure that pulls water from the brain parenchyma [28,29,30]. This interstitial glymphatic drainage thus seems vital to remove toxins from the brain parenchyma and into perivascular, ventricular, and subarachnoid CSF. The CSF flows from ventricles of the brain to the subarachnoid space, and from there, it drains across arachnoid villi and granulations into the superior sagittal sinus; facial and dural lymphatics, including the cribriform plate; and glymphatic pathways across the dura [51,94,95,96,97,98,99,100,101,102,103,104]. Low-pressure lymphatic drainage starts first in the foetus, while the arachnoid pathway has been shown to be an active, energy-requiring, higher-pressure output pathway [100]. Where there is failure to generate enough arachnoid draining cells or a delay in activation, congenital hydrocephalus or external hydrocephalus may result. At any time in life when damage occurs to these drainage cells/pathways through multiple mechanisms, including infection, inflammation, birth asphyxia, and others, drainage capacity may be compromised, resulting in fluid and macromolecule accumulation in the ventricles and external fluid spaces with increasing intracranial pressure. These are associated with a wide range of neurological conditions, as already highlighted with an associated cerebral folate metabolic imbalance. Pathological conditions such as head trauma and neurodegenerative conditions also generate cell-breakdown components and toxins that add to the protein signature of CSF that would result in osmotic changes, adding to the fluid volume building up in the ventricles [28,29,30].

Folate, or vitamin B9, for which folic acid is a synthetic, stable form used in supplements, is a critical co-factor for 1-carbon metabolism. Folate is essential in the synthesis of purines and pyrimidines; for forming DNA and RNA; for nitric oxide, via tetrahydrobiopterin; for cardiovascular and cerebrovascular health; for biogenic amine neurotransmitters; for amino acids, including glycine, serine, cysteine, and glutamic acid; and for glutathione for tissue repair and detoxification and as an antioxidant [33]. These functions make folate an essential dietary requirement for the body, as well as critical to brain development, health, and function throughout life. 5-methyl tetrahydrofolate (5mTHF), the major folate form in nature, in food, and circulating in the body, is essential in the methylation of homocysteine to methionine through the action of vitamin B12 and methionine synthase. High levels of homocysteine are toxic and associated with disease states, including diabetes, heart disease, stroke, and dementia, and decreasing levels can decrease risks for these, as well as improve other conditions [32,33,34,36,105,106,107,108,109]. Methionine generates s-adenosyl methionine (SAM), the universal methyl donor involved in methylation reactions, particularly of DNA and RNA, but also proteins and lipids, crucial for gene regulation and molecular activation/function. Folate thus has a profound impact on health generally, and specifically to brain development, health, and function [7,36,53,110,111,112]. Folate does not work alone and requires additional co-factors in different parts of its metabolic cycle, for example, B12 for methylation of homocysteine; D3 and B2 for the synthesis of the biogenic amine neurotransmitters noradrenaline, dopamine, serotonin, and melatonin, which are key in attention, mood, and sleep disorders; and nitric oxide for cerebrovascular health and autoregulation, as well as B6 and B3 in other metabolic arms [33].

Folate transporters have been noted in the blood–brain endothelial barrier, but these are minimal when compared to the major transport across the blood–CSF barrier, the choroid plexus, by folate receptor alpha (FOLR1), proton-coupled folate transporter (PCFT), and reduced folate carrier (RFC) [113,114,115,116,117,118,119]. Loss of function in FOLR1 results in a cerebral folate deficiency, indicating that this is the major transporter. In knockout mice that are missing FOLR1, RFC increases in expression to transport folate into CSF [120]. The difference in these transporters is that FOLR1 carries folate across the choroid plexus and into CSF, where it then transports folate directly into the brain or together with FDH [117,121]. FDH is significantly reduced or absent in hydrocephalus CSF, leading to a cerebral folate imbalance, poor transport of folate into the developing cortex, and a failure of normal development [35,36]. This is also associated with a significant change in CSF composition orchestrated in part by the reduced FDH [34]. One further cause of cerebral folate deficiency, independent of CSF abnormalities, and identified as a cause of childhood epilepsy and autism, is autoantibodies to FOLR1. Present in neonatal blood, these are maternal antibodies that block FOLR1 transport functions at the choroid plexus [37,38,122,123,124,125]. As an autoimmune disease, this could also occur later in life if FOLR1, rich in certain foods, such as milk, should enter the blood and cause an immune response [14,39,41,44,47,124,126,127].

In this study, we investigated several cerebral neurological/neuropsychiatric conditions that have published associations with enlargement of the lateral and/or third ventricles. We focused on FOLR1, FDH, and folate, as these three molecules would highlight a transport deficiency for folate across the blood–CSF barrier (FOLR1 and folate) and/or a deficiency in access to folate from the CSF by the brain (folate and FDH). FOLR1 is a folate-binding protein that acts as a folate transporter into CSF and cells [117,128,129] existing in both membrane-bound and soluble forms. This protein has also been associated with various neurodegenerative diseases, as it directly affects the transport of folate from both the blood into the cerebrospinal fluid (CSF) and from the CSF into the cerebral cortex [35,121,128,130,131]. Furthermore, FDH, as well as being a key folate enzyme, also has a role similar to FOLR1 in binding to folate for transport into brain cells. Its overexpression in the cerebral cortical cells of animals affected by different congenital neuropathologies, including hydrocephalus, could affect neurodevelopment due to its ability to inhibit cell proliferation in high concentrations [132,133,134]. Additionally, FDH is a marker of a specific set of astrocytes [135] which form a network of cells connected to the CSF and serve as the main folate delivery system in the normal adult brain [32]. An imbalance of or deficiency in folate can occur at any time during development and adult life following a CSF-drainage insufficiency resulting from poor development or through different conditions causing loss of drainage, including infections, inflammation, oxidative stress, head trauma, loss of cells through ageing, etc., or from an autoimmune blockade of FOLR1. These conditions would then result in further effects due to the folate imbalance or deficiency, including, most importantly, cerebrovascular issues, perhaps leading to glymphatic insufficiency, and neurotransmitter imbalance.

Hypothesis and aims: Due to the critical role of CSF in the supply and metabolism of folate for cerebral development and function, cerebral conditions associated with ventricular enlargement are likely to have a folate metabolic error that may underlie their specific functional deficits. Furthermore, although not addressed in the small number of cases analysed in this study, we expect that changes in folate metabolism will reflect the range of ventricular enlargement observed in these conditions, as we have found in the hydrocephalic H-Tx rat, which shows a range of ventricular enlargement correlated with the loss of FDH. This study was therefore designed to analyse cerebral folate status in various cerebral conditions to test this hypothesis. We focused on three key molecules: (i) folate receptor alpha (FOLR1), the transporter for folate from blood to CSF across the choroid plexus (the blood–CSF barrier); (ii) aldehyde dehydrogenase 1L1 (ALDH1L1), also known as 10 formyl tetrahydrofolate dehydrogenase (FDH), the critical enzyme in folate metabolism but also a transporter of folate from CSF into the brain along the CSF pathway; and (iii) folate (vitamin B9), the key metabolite for brain development, health, and function.

## 2. Results

All results are shown as summary graphs of the data. Raw data, including blot images and measurements, are presented in Supplementary Table S1 and western and Supplementary Data S1.

### 2.1. Total Protein

**Figure 1:**
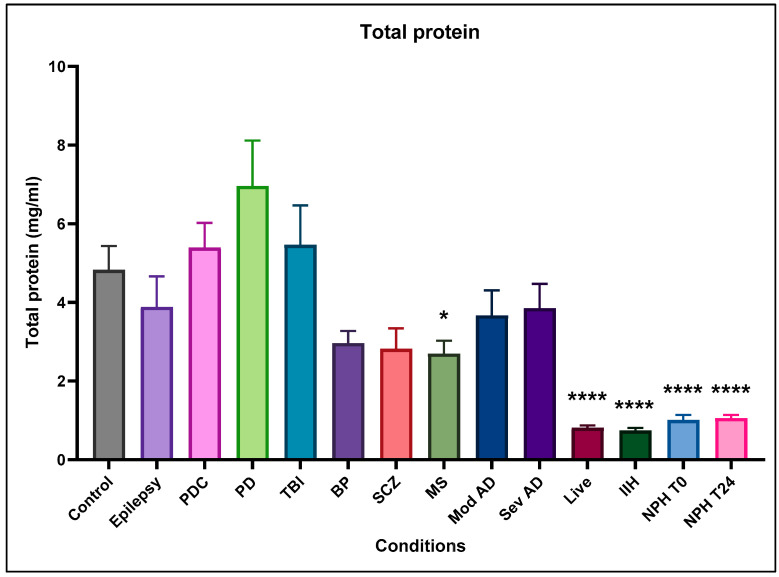
Total protein content of CSF from different neurological conditions. Bars show mean with standard error bar. The proteins values are absolute values, mg/mL, of CSF. Most values are not significantly different from control values. MS, live dementia, IIH, and NPH are all significantly reduced. BP and SCZ are also reduced but not significantly with the number of samples in the study. Data were normalised using the general control sample (non-demented control, 1994-076) added to every Western blot and dot blot. This control sample was set as 1, and measurements from other CSF samples were adjusted according to their intensity relative to the control, including all controls used. * indicates significance at *p* ≤ 0.05, **** indicates significance at *p* ≤ 0.0001.

Compared to normal control CSF, most post-mortem conditions showed no significant difference, although some showed a non-significant reduction in total ventricular protein with Parkinson’s disease (PD) and traumatic injury (TBI), showing a non-significant increase (Figure 1). Multiple sclerosis (MS) CSF showed a significantly decreased protein, and bipolar (BP) and Schizophrenia (SCZ) showed a similar but non-significant decrease. All live-patient samples showed a significantly decreased protein content compared to post-mortem ventricular CSF samples, but these are within the normal range for lumbar CSF. Although the ventricular post-mortem protein levels are high, they are also at the upper end of normal range. The surprising finding is that they are much higher than lumbar CSF, which may be related to the post-mortem time and accumulation of proteins from tissue breakdown and/or lack of use (see also Discussion section). Post-mortem times ranged from 3.5 to 155 h (see Supplementary Materials Table S1). With the heart and respiration stopped, there would be zero pulsatility in the CSF to mix and move the CSF and its contents so that accumulation of proteins from the choroid plexus, from brain interstitial fluid, and from tissue breakdown may be substantial. Using a simple Pearson r correlation for all the post-mortem samples, we found a moderate positive correlation between post-mortem time and total protein (*p* = 0.35) but no correlation with FDH, FOLR1, or folate concentrations.

### 2.2. Folate

**Figure 2:**
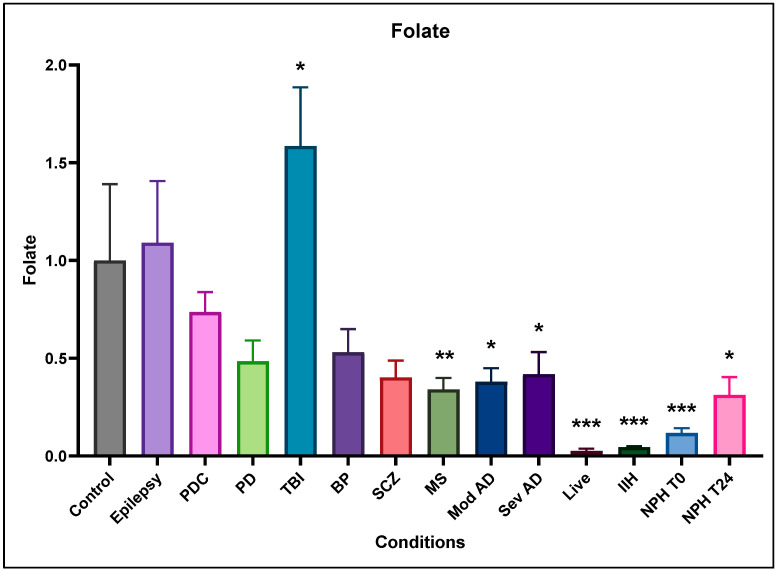
Relative (to the control) concentration of folate in CSF from the different neurological conditions. Epilepsy is not different from control, but PDC, PD, BP, and SCZ all show a non-significant decrease, while MS, AD, live dementia, IIH, and NPH all show significantly reduced folate levels. TBI is unique in showing a significantly increased folate concentration in CSF. Data were normalised using the general control sample (non-demented control, 1994-076) added to every Western blot and dot blot. This control sample was set as 1, and measurements from other CSF samples were adjusted according to their intensity relative to the control, including all controls. * indicates significance at *p* ≤ 0.05, ** indicates significance at *p* ≤ 0.01, *** indicates significance at *p* ≤ 0.001.

Folate was reduced in all conditions except epilepsy and mild traumatic brain injury (Figure 2). Compared to control post-mortem ventricular CSF levels, epilepsy and Parkinson’s disease controls (PDCs) showed no significant difference from the controls. Parkinson’s, bipolar (BP), and Schizophrenia (SCZ) showed a non-significant reduction in folate, while multiple sclerosis (MS) and moderate (Mod AD) and severe (Sev AD) Alzheimer’s had significantly reduced folate. Samples from live dementia (Live), intracranial hypertension (IIH), and normal-pressure hydrocephalus (NPH) patients much greater significance in regard to their reduced folate levels. Interestingly, at the end of their 24 h lumbar CSF drain, CSF folate levels were higher at the end of the drain (NPH T24 vs. NPH T0), suggesting that NPH patients suffer a cerebral folate deficiency as a consequence of the condition. Mild traumatic brain injury (TBI) samples were unique in showing a significant elevation in folate levels.

### 2.3. Folate Receptor Alpha (FOLR1)

**Figure 3:**
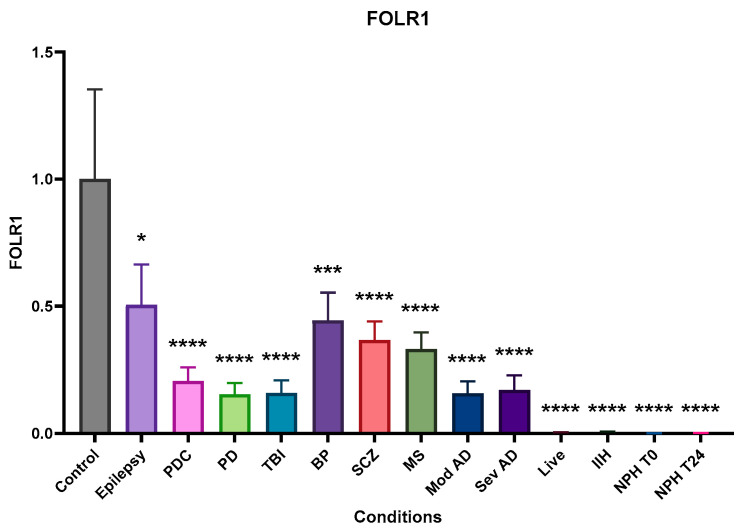
Relative (to the control) concentration of folate receptor alpha (FOLR1) in CSF from the different neurological conditions. All conditions show significantly reduced FOLR1 levels with barely detectable levels in the LP CSF of live dementia, IIH, and NPH. Bars are means plus SEM. Data were normalised using the general control sample (non-demented control, 1994-076) added to every Western blot and dot blot. This control sample was set as 1, and measurements from other CSF samples were adjusted according to their intensity relative to the control, including all controls used. * indicates significance at *p* ≤ 0.05, *** indicates significance at *p* ≤ 0.001 **** indicates significance at *p* ≤ 0.0001.

All conditions showed a significant reduction in FOLR1 compared to control post-mortem CSF, with live patients having practically undetectable levels (Figure 3). These essentially following trends in folate concentrations, but TBI is notable in that it has significantly higher folate than the control but very low FOLR1. In addition, while folate levels increase in T24 NPH CSF compared to T0, FOLR1 does not increase.

### 2.4. 10-Formyl Tetrahydrofolate Dehydrogenase (FDH and ALDH1L1)

**Figure 4:**
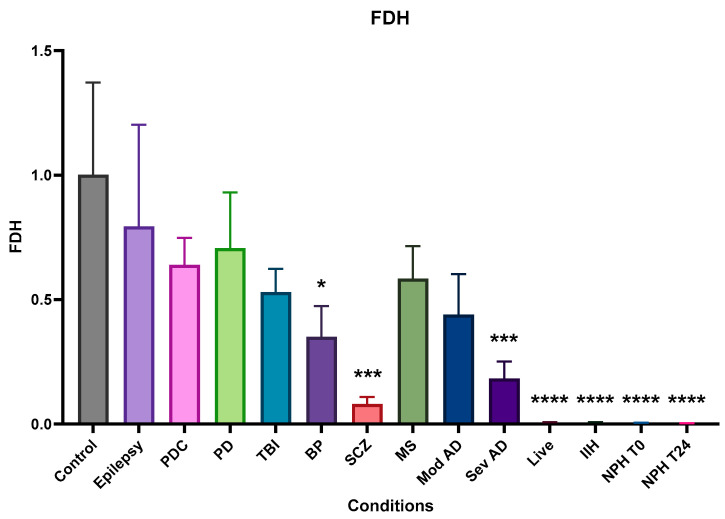
Relative (to the control) concentration of FDH in CSF from the different neurological conditions. Epilepsy, PDC, PD TBI, MS, and moderate AD have no significant difference from control levels of FDH in CSF. BP, SCZ, and severe AD have significantly reduced levels of FDH in CSF, while live dementia, IIH, and NPH have barely detectable levels in LP CSF. Bars are means plus SEM. Data were normalised using the general control sample (non-demented control, 1994-076) added to every Western blot and dot blot. This control sample was set as 1, and measurements from other CSF samples were adjusted according to their intensity relative to the control, including all controls used. * indicates significance at *p* ≤ 0.05, *** indicates significance at *p* ≤ 0.001 **** indicates significance at *p* ≤ 0.0001.

All conditions showed some reduction in FDH, but only SCZ; Sev AD; and live patients with dementia, IIH, and NPH showed very significant reductions in FDH (Figure 4). BP showed a less significant reduction, while the remaining conditions showed non-significant reductions.

## 3. Discussion

**Figure 5:**
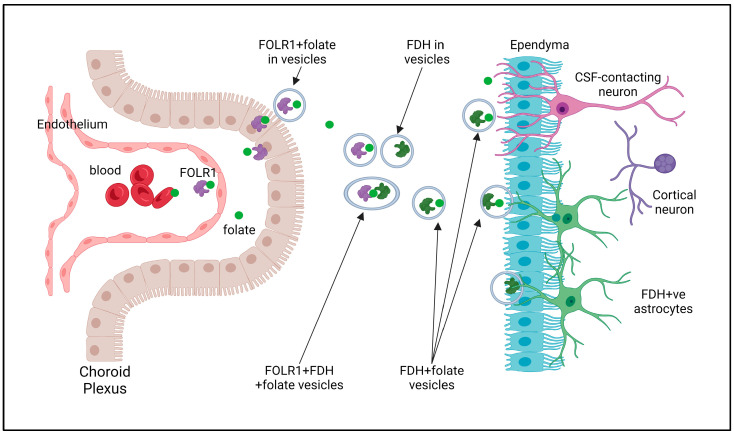
Schematic diagram (created using BioRender.com/o82e774) illustrating proposed route of folate supply to the cerebral cortex. Folate is transported in blood, within erythrocytes and plasma bound to FOLR1 and folate-binding proteins. In the choroid plexus, fenestrated capillaries allow FOLR1 and free folate (and other blood components) to enter the interstitial space on the basal side of the choroid epithelium. Here, membrane-bound FOLR1 accepts free folate, while folate bound to FOLR1 inserts into the membrane. FOLR1 then invaginates to release folate into the cell and then merges with the apical membrane to produce vesicles containing FOLR1 and folate that are sent into the CSF. FDH is synthesised in radial glial and astroglial cells that send vesicles of FDH into the CSF. These fuse with the FOLR1–folate vesicles to transfer folate from FOLR1 to FDH. FDH–folate can then enter the cortex through the GFAP-negative–FDH-positive astroglial network. Folate may also enter directly from CSF into CSF-contacting neurons. Folate is transported around the CSF pathway as free folate and bound to FDH, and this is important to supply the grey matter with folate through the subarachnoid CSF and pial interface as well as the meninges.

Figure 5 is a schematic illustrating how we believe folate is transported into the CSF and onward into the brain. Folate is carried around the body through the blood in erythrocytes, where it is bound to FOLR1; FOLR1 also exists in blood plasma along with free folate and folate bound to other proteins. Fenestrated capillaries in the choroid plexus allow for the exit of folate and FOLR1 into the interstitial space, and from there, FOLR1 binds to the choroidal membrane, invaginates, and transports folate across the choroid and out into the CSF in vesicles [117,121,129]. FOLR1 transports folate around the CSF pathway and also transfers it to FDH via the fusion of their respective vesicles as FDH enters the CSF from the brain in vesicles/cellular fragments [35]. Transfer to FDH is required for folate transport into the brain’s uptake pathway. This is into FDH-positive astrocytes that form a network throughout the cortex [32]. Folate can also enter with FOLR1 into GFAP-positive astrocytes, and this process becomes important in abnormal conditions such as Alzheimer’s disease [35]. FDH also transports folate throughout the CSF pathway and is important in delivering folate to the cortex from the subarachnoid space [32].

**Figure 6:**
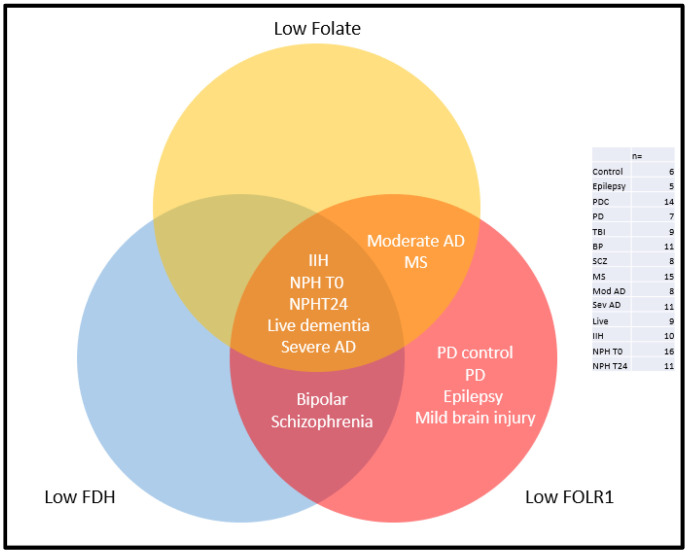
Venn diagram illustrating the changes in FOLR1, folate, and FDH concentrations in the CSF of the conditions investigated in this study. No condition was normal. PDC, PD, epilepsy, and TBI had reduced FOLR1 but normal FDH and folate, indicating normal folate transport into the CSF but that folate may be entering the brain with FOLR1 rather than FDH, as we have described in severe AD [28]. Bipolar and SCZ have reduced FDH and FOLR1 but normal folate, suggesting a problem regarding transport into CSF, as well as into the brain. Moderate AD and MS have reduced folate and FOLR1, indicating a significant obstruction to folate transport into the CSF. IIH, NPH, live dementia, and severe AD have a reduction in all three molecules, indicating a profound cerebral folate deficiency. The table indicates the number of cases analysed for each condition.

The aim of the current research was to test whether cerebral neurological conditions that have published associations with ventricular enlargement also have a cerebral folate metabolic issue affecting brain health and function. This hypothesis was based on studies of neonatal and congenital hydrocephalus in which we found an obstruction in access to CSF folate through a blockade of FDH release into CSF. Although the conditions we analysed appear very different, they share some common features, including a severity association with the increasing size of the cerebral ventricles [1,2,3,4,5,6,7,8,9,10,11,12,13,14,15,16,17,18,19,20,21,22,23,24,25,26,27]. Even with the limited number of cases examined, the data lend support to the hypothesis, showing that all of the conditions examined do have an associated issue in folate transport from blood into CSF, from CSF into the brain, and/or in folate concentration (Figure 6).

The Venn diagram (Figure 6) illustrates the main findings. Confirming our original hypothesis, no condition was wholly “normal” in the folate profile when compared to normal age-matched controls with no known neurological condition. IIH, NPH, live dementia, and severe Alzheimer’s (Braak V-VI) CSF showed significant reductions in all three molecules, indicating a profound cerebral folate deficiency. Moderate Alzheimer’s disease (Braak III-IV) and MS CSF had significantly reduced folate and FOLR1 but normal FDH, indicating a possible failure in folate transport from the blood into the CSF but no loss of FDH from the brain. PD, epilepsy, and mild traumatic brain injury showed a significant reduction in FOLR1 but not in folate or FDH, indicating successful folate transfer from blood and into the brain and potential recycling of FOLR1 or its use in transport into the brain, as we have described before for Alzheimer’s disease [32]. BP and SCZ showed significant reductions in FDH and FOLR1 but not folate, suggesting successful transport of folate across the blood–CSF barrier but a failure of normal transport into the brain due to low FDH, which we have previously described in Alzheimer’s disease and hydrocephalus. The low FOLR1 would indicate that folate may be transported into the brain using FOLR1, similarly to that seen in Alzheimer’s [32], and this finding might also indicate a similar change in metabolism to that seen in Alzheimer’s, as well. In Alzheimer’s, we saw a failure of folate uptake via the FDH pathway, but FOLR1–folate entered via GFAP + ve astrocytes, supplying neurons directly, and this was associated with the hypermethylation of neuronal nuclei, suggesting a shutdown of function as part of the pathology in severe Alzheimer’s disease [32,33].

Given the critical role of folate in DNA synthesis, methylation, neurotransmitter synthesis, and nitric oxide production, abnormalities in folate metabolism would have serious consequences to any/all of these functions, as well as to associated metabolic processes reliant on the products of folate metabolism. We have already described a significant change in the folate delivery pathway, as well as a change in metabolism to hypermethylation in the Alzheimer’s cortex [32,33]. The timing of insult and resulting specific imbalance in folate metabolism found in these cerebral neurological conditions could explain the specific functional effects, as well as the shared functional effects, seen clinically. Most notably, effects on cerebrovascular function, autonomic functions, and motor functions are common across these conditions, while mild-to-severe dementia also manifests in some of these conditions, notably in NPH, Parkinson’s, and head trauma. Many of these co-morbidities have not previously been linked together, but it seems likely that a folate fault could explain them in these varied conditions. Investigations of the specific faults in each condition have already revealed a profound cerebral folate deficiency in normal-pressure hydrocephalus, only partially rescued by CSF drainage (Ikeda et al., in preparation); a shift in the folate delivery pathway and metabolism in Alzheimer’s disease, leading to hypermethylation of cerebral neurons [32]; and a specific block in the folate–tetrahydrobiopterin pathway to dopamine synthesis in Parkinson’s disease (Doddapallapur et al., in preparation). It seems highly likely that we may find specific effects on folate metabolism in all of the conditions with an associated enlargement of the cerebral ventricles, with the severity of the effect on metabolism mirroring the severity of fluid accumulation.

Some issues in the data include the comparison of ventricular CSF from post-mortem samples with lumbar-puncture CSF from live-patient samples. Normally, LP samples in live patients have significantly more protein compared to ventricular CSF [136,137], but, in our samples, ventricular CSF from post-mortem brains had more protein than live LP samples. Both fall within the reported normal ranges, so the relative increase in ventricular CSF protein may be due to the post-mortem accumulation of protein, as well as through cell death around the CSF. This may also support theories that the CSF within and around the cerebral cortex does not directly mix with the CSF in the space around the spinal cord. Mixing may occur through blood and respiratory pulsations, as well as through spinal movements, all of which are absent after death. There is evidence in some studies of preferential targeting of lumbar CSF in conditions affecting the lumbar spinal cord or spine [136,137]. The exact interactions between cerebral and lumbar CSF remain in contention and the subject of speculation, theory, and modelling, but it is clear that there are differences in the distribution of key molecules. It is possible that CSF remains a physiological fluid also for the spinal cord, even if, in humans, the central canal becomes closed in adults. We believe, therefore, that although raised, the molecules of interest in our analysis remain in physiological levels in post-mortem samples in the ventricles and are reflected in the living LP samples. Furthermore, our live NPH T0 and T24 samples show little changes in the key molecules, except in showing an increase in folate at T24. The T24 samples followed a 24 h drain of 200 mL of CSF that should have equalised the LP CSF samples with CSF from ventricles/subarachnoid space. The fact that there is little change in the key molecules in T24 CSF indicates, perhaps, that physiologically important molecules are maintained at correct levels, while total protein may change. This requires further analyses. Future tissue analyses will also help to define abnormalities in folate transport into the brain and consequences on folate metabolism and cellular functions. These studies are ongoing.

## 4. Materials and Methods

### 4.1. Ethics

All human tissues were acquired from registered human brain tissue banks in the UK and Netherlands. UK brain banks are regulated under the UK Human Tissues Act 2004, requiring informed consent from donors prior to being deceased. Similarly, the Netherlands Brain Bank operates under local laws and regulations and adheres to the ethical standards of BrainNet Europe’s code of conduct for brain banking (www.brainbank.nl/about-us/ethics; accessed on 19 July 2022). HTA oversight of acquisition and use of human tissue occurred through the Institutional Research Governance, Ethics and Integrity Officer (Human Tissues), at the University of Manchester.

### 4.2. Tissue Supply

Post-mortem human CSF samples from donors suffering from epilepsy, Schizophrenia, and bipolar, as well as normal controls with no neurological condition, were obtained from the Netherlands Brain Bank. Post-mortem Alzheimer’s disease and non-demented controls, as well as traumatic head-injury CSF samples, were obtained from the Manchester Brain Bank, while Parkinson’s disease and multiple sclerosis samples, along with controls, were obtained from the Multiple Sclerosis and Parkinson’s Tissue Bank at Imperial College London. CSF from living human patients with normal-pressure hydrocephalus (NPH) was supplied by the Royal Preston Hospital Neurosurgery Department as part of its clinical research project. NPH patients received a 200 mL lumbar CSF drain over 24 h to test for symptomatic relief. CSF samples were collected at the start (T0) and end (T24); the latter was categorised as responders or non-responders depending on symptomatic relief. CSF samples from living dementia and idiopathic intracranial hypertension patients were obtained from the Walton Centre Biobank in Liverpool. All CSF samples were collected and frozen at −80 °C by the different source biobanks. Samples were transported to Manchester on dry ice, aliquoted into smaller volumes, and then stored frozen at −80 °C until analysed. The number of patients in each condition and their source are summarised in Table 1. All patient data and samples were received anonymised but with key clinical data included, as detailed in Supplementary Materials Table S1. Post-mortem samples had post-mortem delays in removing and preserving tissue ranging from 3.5 to 155 h, giving significant time for protein accumulation in CSF compared to the live samples. A larger sample size would allow for a correlation of protein levels to post-mortem delays.

### 4.3. Western and Dot Blotting

For Western blotting, aliquots were diluted 2:1 with 2× NuPAGE^TM^ LDS Sample Buffer (Thermo Fisher, Paisley, UK), to which 5% MercaptoEthanol (mEtOH, Sigma-Aldrich, Poole, UK) was added. This sample mix was heated to 90 °C for 5 min to denature and reduce the proteins to their 1D structure to facilitate electrophoresis. Then, 9 µL of each sample was loaded into NuPAGE^TM^ 4–12% Bis-Tris 12-well ready-gels running in 500 mL of 1× NuPAGE^TM^ MES running buffer (Thermo Fisher, Paisley, UK). Gels were run at 120 V for 1 h. Gels were extracted from the plastic running plates and placed onto the nitrocellulose membrane of the Invitrogen^TM^ Power Blotter Select Transfer stacks (Thermo Fisher, Paisley, UK), which, after reassembly, were placed into the Invitrogen^TM^ Power Blotter Station (Thermo Fisher, Paisley, UK) for protein transfer from the gels to the membrane for 10 min, at 25 V, 1.3 A. For dot blots and total protein analysis, samples were analysed without any pre-treatment. Then, 2 µL of CSF was pipetted directly onto dry nitrocellulose membranes and allowed to dry. Membranes from western and dot blots were stained using the Invitrogen iBind system (Thermo Fisher, Paisley, UK), with both primary and secondary antibodies (Table 2), by placing the membrane face-down onto the iBind card pre-soaked with 1× iBind flex buffer. Antibodies were added to 2 mL of iBind flex buffer (Rabbit anti-FOLR1 at 1:1000; Rabbit anti-FDH at 1:2000; and 1:4000 dilution for the StarBright Blue 700 Goat anti-Rabbit secondary). Antibody specificity was tested against the partial proteins used as the antigen by the manufacturer, as well as using positive controls, known to contain significant concentrations of the target molecule from previous studies, as well as negative controls (e.g., hydrocephalus samples), known to have undetectable levels of the target protein. Antibodies were bought from companies with in-house validation of target specificity, giving a double assurance of antibody specificity. The membrane remained in the iBind device until the entire buffer and antibody volumes were completely empty from the starting wells and/or left overnight to complete the process. Completed membranes were scanned with the Bio-Rad ChemiDoc MP imager (Bio Rad Laboratories Ltd., Watford UK) at the secondary antibody wavelength. Scans were visualised and analysed using Bio-Rad Image Lab software, version 6.1. Antibody specificities and specific band location were demonstrated by performing a blot with positive and negative controls. All protein bands were normalised to a standard control run in every gel. All samples were run at least 3 times each, on different gels. All gel and dot blot images are presented in the Supplementary Data S1. Raw data are presented in Supplementary Table S2, with summary data in the results. Positive controls used the partial protein antigens used by the companies that manufactured the antibodies (Table 2 and Table 3). Negative controls involved excluding the primary antibody.

### 4.4. Total Protein Measurement

For total protein analysis, 2 μL of raw sample was scanned using the NanoDrop^TM^ 2000 Spectrophotometer (Thermo Fisher, Paisley, UK) at a wavelength of 280 nm, which detects total protein. Three aliquots of each sample were scanned. Data are shown in Supplementary Table S2 and are plotted as actual protein concentrations.

### 4.5. Data Analysis

Three aliquots of each sample were analysed three times each for the same parameter, and the average of each sample was used for the analysis. For both the dot blot and Western blot, the intensity signal was measured after background subtraction, using the Bio-Rad Image Lab 6.0 software. Data were normalised to a control sample that was added to every gel. Data were analysed using ANOVA in GraphPad Prism software, version 10. Outliers, excluded from the analysis and highlighted in the Supplementary Materials’ data table, were defined as having an average value 5 times or greater than the median of the group. Outliers and samples which were not used in all the measurements were excluded from all 4 measurements and the analyses.

## 5. Conclusions

CSF is a physiological fluid that is key to cerebral cortex development, function, and health. One of the essential functions of CSF is folate supply and metabolism for the brain, including maintaining metabolic balance. Where CSF circulation and/or drainage becomes abnormal, indicated by fluid accumulation and ventricular enlargement, this can affect the balance of folate metabolism, as well as the supply of folate. Pathway effects can include transport of folate into the CSF, across the choroid plexus with FOLR1; transfer to FDH for direct transfer to the brain; and FOLR1/FDH folate transport around the CSF pathway, particularly to the subarachnoid space and pial surface of the cortex. These changes are determined according to the time and severity of the insult and/or the progression of the condition or disease. These, in turn, determine the specific imbalance that occurs in folate metabolism and the pathways dependent upon this. This study lays the foundation to investigate the cerebral conditions affected based on this hypothesis and to better understand the metabolic disorders underlying them. In particular, folate is essential to nitric oxide synthesis, so cerebrovascular failures as cause and implicated in progression may be operating. Folate is also essential for neurotransmitter synthesis via tetrahydrobiopterin, so transmitter imbalance/deficiencies may also be caused by a folate imbalance. Epigenetic changes, through changes in methylation, can also be mediated through folate disruption and failure to generate s-adenosyl methionine (SAM), the universal methyl donor. The resulting abnormal gene expression would result in malfunctions in multiple systems. In severe Alzheimer’s disease, we have found a shutdown of gene expression and potential protection of neurons through hypermethylation that is mediated through a change in the folate supply pathway and metabolic balance [32,33]. Given the essential role of folate in brain development, function, and health, as well as multiple metabolic pathways dependent on its products, it seems that even small changes in folate metabolism may have significant consequences for brain function. Cerebral folate status is protected from fluctuations in blood folate supply through active transport mechanisms that maintain a normal concentration around four times that in the blood. But cerebral folate is extremely sensitive to CSF integrity, flow, and drainage. In foetal-onset hydrocephalus, for example, we found that the FDH release from cortical radial glia was inhibited at the onset of CSF high-volume output, i.e., before any possible rise in pressure, indicating an exquisite mechanism to stop folate supply to the developing cortex if any abnormality in CSF drainage occurs. Early events, perhaps related to oxidative stress, insults/trauma, or other factors, resulting in even minor changes in CSF drainage could result in such changes, leading to metabolic consequences, as we have found in the cerebral conditions. This clearly requires further detailed investigations, which we are undertaking.

## Figures and Tables

**Table 1:** Conditions, source, and number (*n*).

Condition	(*n*)	Source
Control	7	Netherlands Brain Bank
Epilepsy	6	
Schizophrenia (SCZ)	12	
Bipolar disorder (BP)	13	
Non-Parkinson’s (PDC)	14	Imperial College Brain Bank
Parkinson’s disease (PD)	9	
Multiple sclerosis (MS)	15	
Mild traumatic brain injury (TBI)	9	Manchester Brain Bank—brains for dementia research
Moderate Alzheimer’s (AD)	10	
Severe Alzheimer’s (AD)	12	
Live dementia	9	Liverpool Walton Centre Biobank
Intracranial hypertension (IIH)	10	
Normal-pressure hydrocephalus T0	14	Preston Royal Hospital
and T24	13	

**Table 2:** Antibodies used.

Primary Antibody	Cat. Number	Source	Host Species	Working Concentration
Polyclonal anti-ALDH1L1	17390-1-AP	Proteintech	Rabbit	1:2000
Polyclonal anti-FOLR1	29472-1-AP	Proteintech	Rabbit	1:2000
Monoclonal anti- 5MTH folic acid	M5028	Sigma-Aldrich	Mouse	1:2000
Secondary Antibody				
StarBright Blue 700 Goat anti-Rabbit IgG	12004162	BIORAD	Goat	1:4000
StarBright Blue 700 Goat anti-Mouse IgG	12004158	BIORAD	Goat	1:4000

**Table 3:** Positive controls used.

Protein	Source	Cat. Number	Info
FOLR1	Proteintech	Ag19959	GST fusion protein—partial length
ALDH1L1	Abnova	H00010840-q01	60Kd partial protein

## Data Availability

All raw data is available from the corresponding author.

## Supplementary Materials

The following supporting information can be downloaded at: https://www.mdpi.com/article/10.3390/ijms251810205/s1.
