# Supplementary material for: Accumulation of Cerebrospinal Fluid, Ventricular Enlargement, and Cerebral Folate Metabolic Errors Unify a Diverse Group of Neuropsychiatric Conditions Affecting Adult Neocortical Functions
Source: Int J Mol Sci. 2024 Sep 23;25(18):10205. doi: 10.3390/ijms251810205 (PMC11432090; doi:10.3390/ijms251810205)
Supplement: Supplementary file 1 [file ijms-25-10205-s001.zip › Supplementary Data S1 blot images.pdf]

Supplementary Data s1 western and dot blot images

Images of western blots

The sequence of samples is given in the table below each image numbered left to right with the left always loaded next to the molecular weight marker. The control of controls is included in every western and dot blot for comparisons across blots..

The red arrowhead marks 100kD where the ALDH1L1 band is expected. The blue arrowhead marks 37kD where the FOLR1 band is expected.

Blank cells in the tables show empty wells.

Cells with NR written in the tables were wells loaded with non-relevant samples.

Control and Multiple Sclerosis

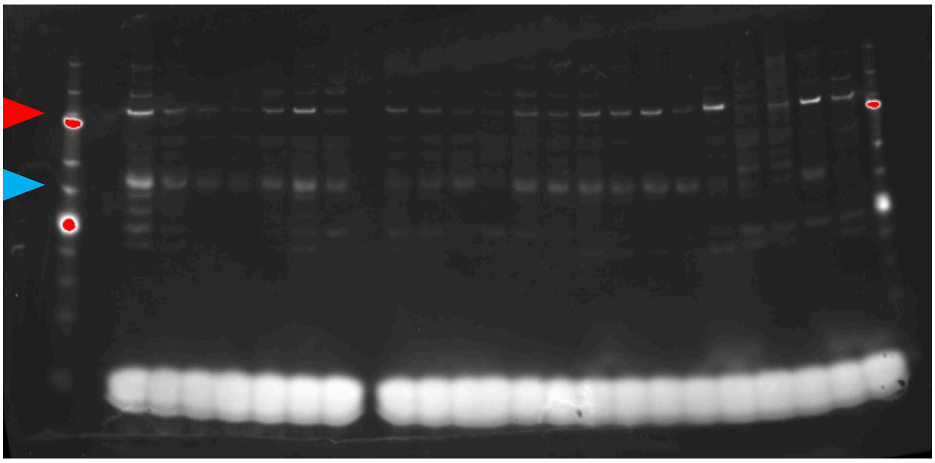

	1	2	3	4	5	6	7	8	9
1	Marker		93-012	92-030	92-026	90-059	92-013	90-019	94-760
2		MS528	MS411	MS407	MS387	MS199	MS125	MS115	MS086
3	MS071	MS061	MS049	MS547	MS543	MS026	MS023	Marker	

Supplementary Data s1 western and dot blot images

Bipolar, Epilepsy, Schizophrenia -1

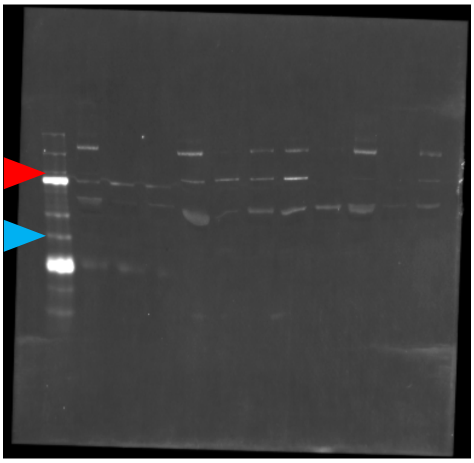

Marker	1994-076	NR	2000-088	2002-045	2010-055
NR	NR	2015-069	NR	NR	2006-075

Bipolar, Epilepsy, Schizophrenia -2

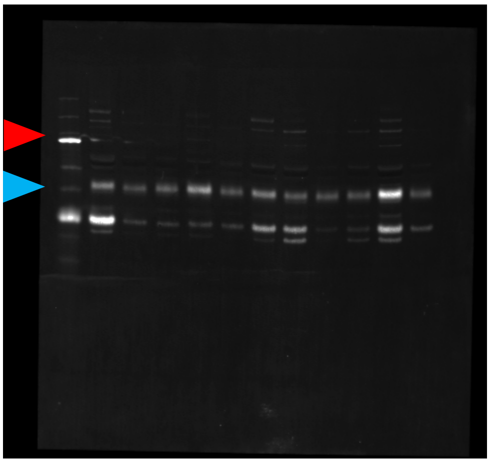

Marker	1994-076	2000-111	2008-081	2012-127	NR
2010-087	2014-070	2007-076	2014-041	1993-143	2016-062

Supplementary Data s1 western and dot blot images

Bipolar, Epilepsy, Schizophrenia -3

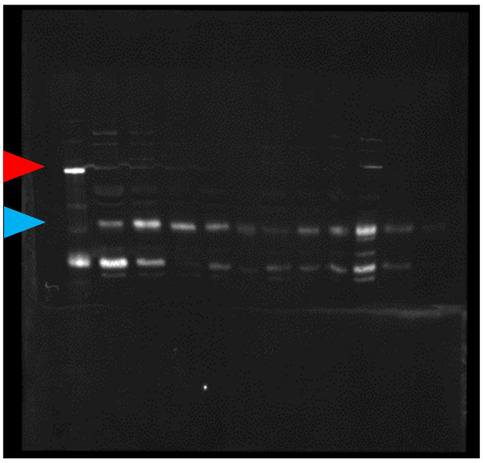

Marker	1994-076	NR	2013-038	2018-102	2010-127
2015-077	2016-003	2015-031	NR	2015-059	1997-134

Bipolar, Epilepsy, Schizophrenia -4

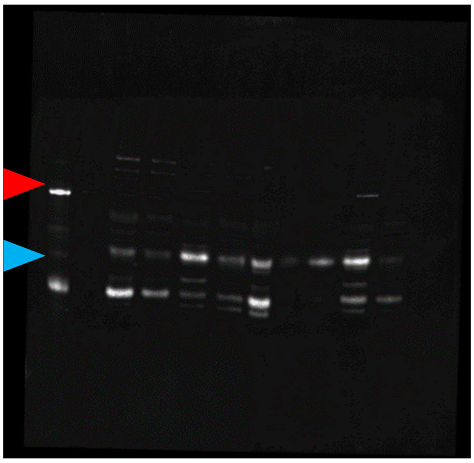

Marker		1994-076	2013-006	2015-044	2004-004
2010-021	2012-048	2005-046	1995-074	2015-093	

Supplementary Data s1 western and dot blot images

Live dementia

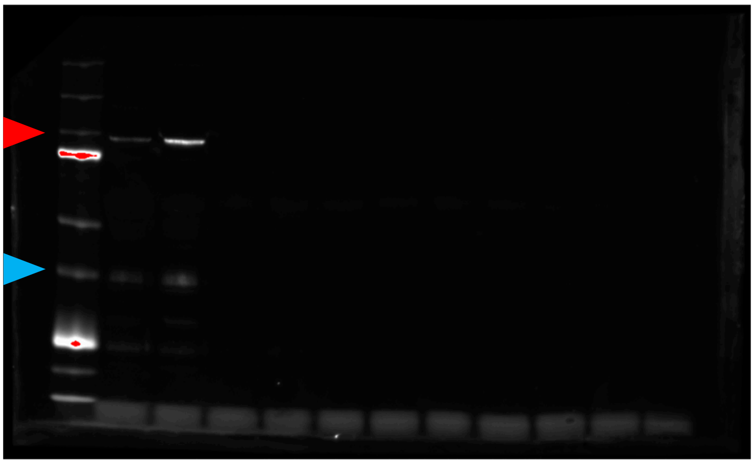

Marker	94-076	93-012	LW1	LW2	LW3
LW4	LW5	LW6	LW7	LW8	LW9

Traumatic Brain Injury

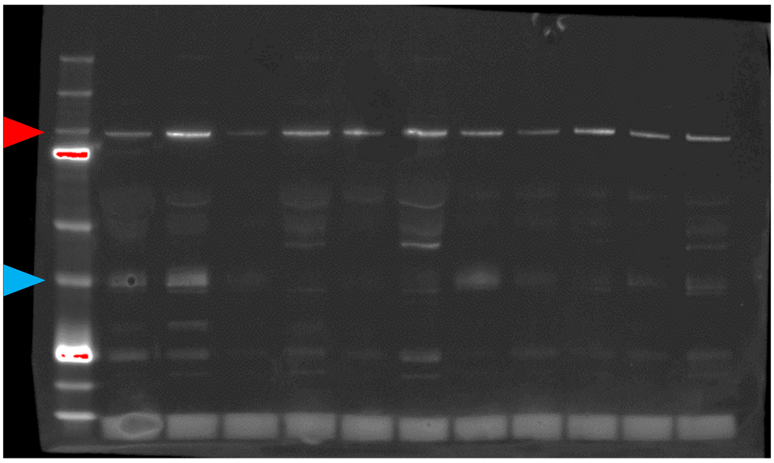

Marker	94-076	93-012	HI 11/28	HI 14/34	HI 16/11
HI 16/31	HI 17/06	HI 17/23	HI 18/29	HI 18/35	HI 18/39

Supplementary Data s1 western and dot blot images

Intracranial hypertension

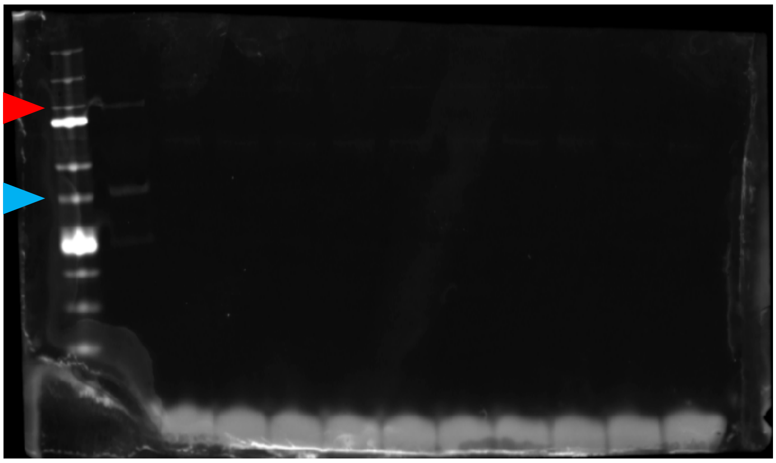

Marker	94-076	IIH093	IIH006	IIH028	IIH058
IIH209	IIH062	IIH007	IIH009	IIH036	IIH208

Normal Pressure Hydrocephalus T0, T24 -1

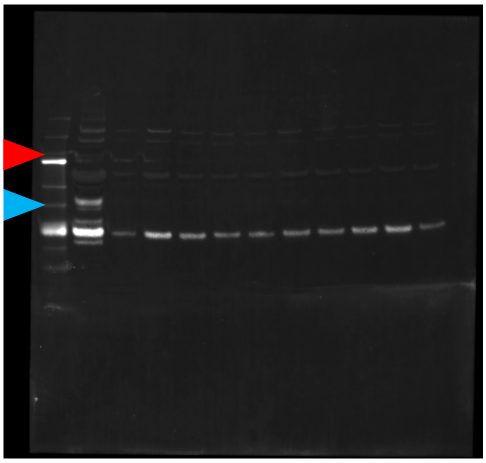

Marker	94-076	T0 040	T0 058	T0 013	T0 044
T0 052	T24 052	T24 049	T24 041	T24 004	T24 045

Supplementary Data s1 western and dot blot images

Normal Pressure Hydrocephalus T0, T24 -2

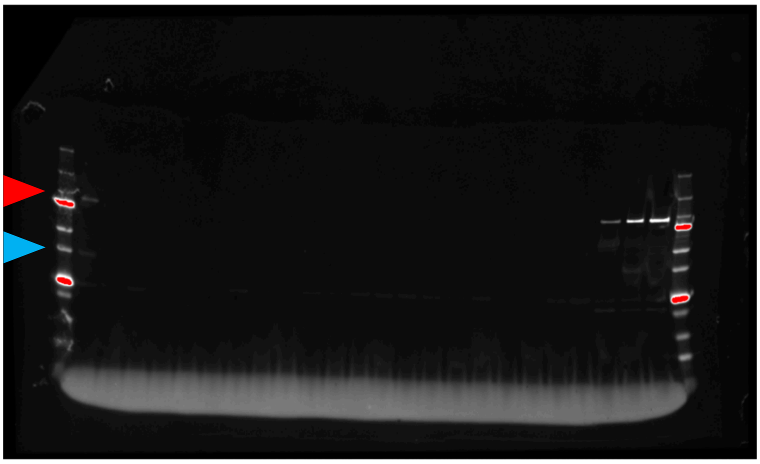

Marker	94-076	T0 004	T0 057	T0 041	T0 001	T0 003	T0 025	T0 033
T0 045	T0 049	T0 056	T0 014	T24 057	T24 044	T24 001	T24 003	T24 025
T24 033	T24 052	T24 056	T24 014	NR	NR	NR	Marker	

Moderate and severe Alzheimer's disease

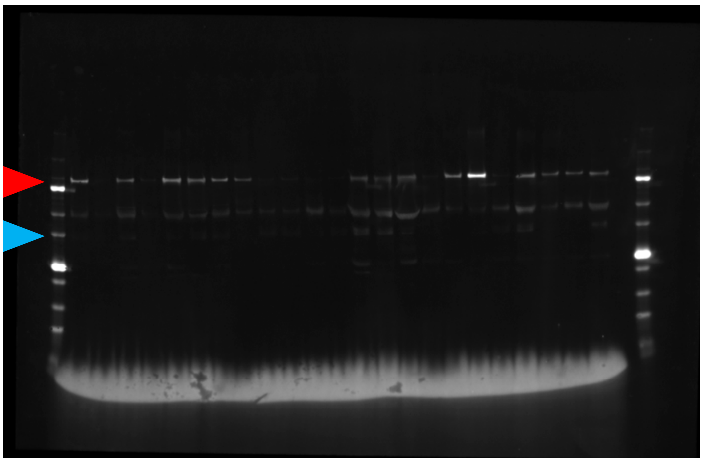

Marker	94-076		DPM11/09	DPM12/34	DPM13/30	DPM14/18	DPM14/35	DPM15/46
DPM16/36	DPM16/37	DPM17/28	DPM11/28	DPM12/01	DPM12/25	DPM13/10	DPM14/07	DPM14/10
DPM14/30	DPM14/31	DPM14/50	DPM15/02	DPM16/10	DPM18/27		Marker	

Supplementary Data s1 western and dot blot images

Parkinson's disorder

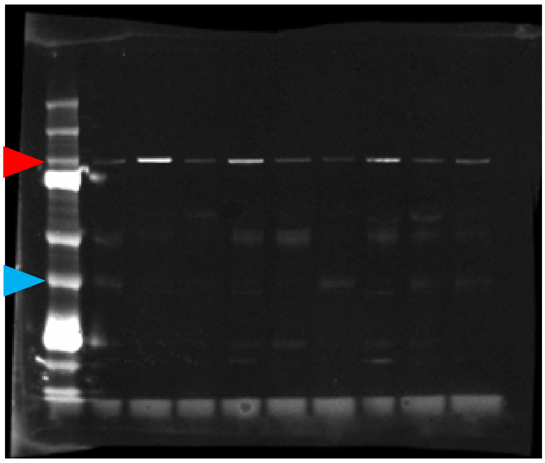

Marker	94-076	PD1209	PD1044	PD1219	PD1221
PD1039	PD1217	PD1216	PD100		

Non-Parkinson's

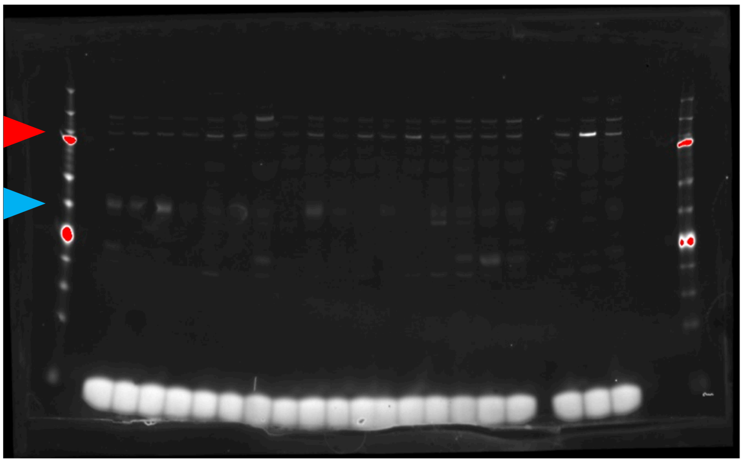

Marker		94-760	NR	NR	PDC030	PDC033	PDC035	PDC052
PDC059	PDC067	PDC068	PDC069	PDC078	PDC084	PDC114	PDC126	PDC128
	PDC131		NR	NR	NR			Marker

Supplementary Data s1 western and dot blot images

Images of dot blots for folate

Control, Bipolar, Epilepsy, Schizophrenia

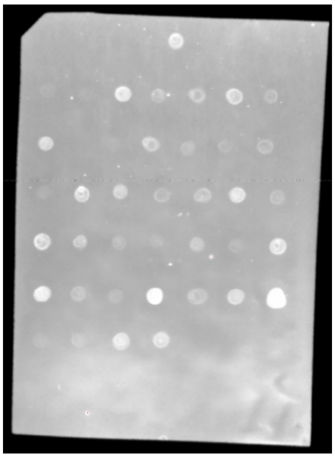

Top \ left	1	2	3	4	5	6	7	(right)
1	1994-076							
2	1992-026	2000-088	2002-045	2010-055	1992-030	1990-019	2015-069	
3	1994-076		1990-059	2006-075	2000-111	2008-081	2012-127	
4	2012-031	2010-087	2014-070	2007-076	2014-041	1993-143	2016-062	
5	1990-031	2013-038	2018-102	2010-127	2015-077	2016-003	2015-031	
6	1993-012	2015-059	1997-134	2013-006	2015-044	2004-004	2010-021	
7	2012-048	2005-046	1995-074	2015-093				
(bottom)								

Supplementary Data s1 western and dot blot images

Live dementia, Traumatic Brain Injury, Intracranial hypertension

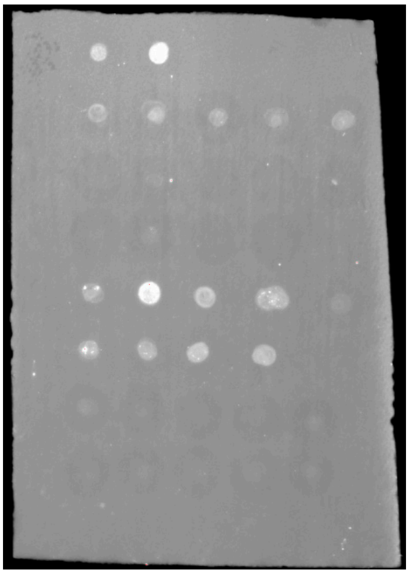

	(left)	1	2	3	4	5	(right)
**(top)**							
1		94-076	NR				
2		NR	NR	NR	NR	NR	
3		LW1	LW2	LW3	LW4	LW5	
4		LW6	LW7	LW8	LW9		
5		HI 11/28	HI 14/34	HI 16/11	HI 16/31	HI 17/06	
6		HI 17/23	HI 18/29	HI 18/35	HI 18/39		
7		IIH093	IIH006	IIH028	IIH058	IIH209	
8		IIH062	IIH007	IIH009	IIH036	IIH208	
(bottom)							

Supplementary Data s1 western and dot blot images

Multiple Sclerosis

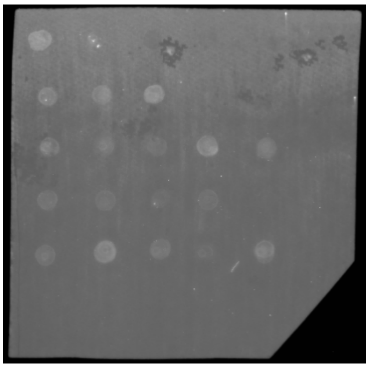

	(left)	1	2	3	4	5	(right)
**(top)**							
1		NR	NR	NR	NR		
2		NR	NR	94-760			
3		MS528	MS411	MS407	MS387	MS199	
4		MS125	MS115	MS086	MS071	MS061	
5		MS049	MS547	MS543	MS026	MS023	
(bottom)							

Normal Pressure Hydrocephalus T0, T24 -1

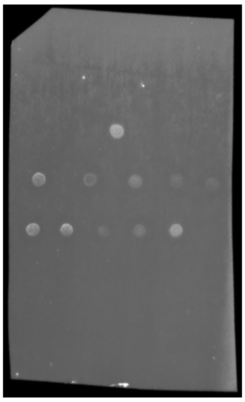

	(left)	1	2	3	4	5	(right)
**(top)**							
1		94-076					
2		T0 040	T0 058	T0 013	T0 044	T0 052	
3		T24 052	T24 049	T24 041	T24 004	T24 045	
(bottom)							

Supplementary Data s1 western and dot blot images

Normal Pressure Hydrocephalus T0, T24 -2

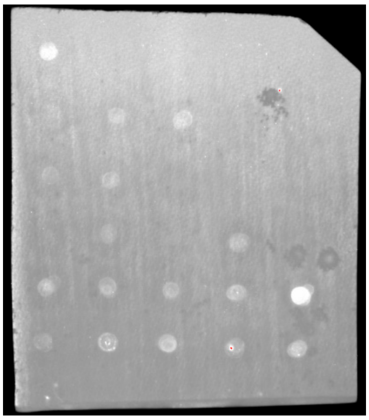

	(left)	1	2	3	4	5	(right)
(top)							
1		94-076					
2		T0 004	T0 057	T0 041	T0 001		
3		T0 003	T0 025	T0 033			
4		T0 045	T0 049	T0 056	T0 014		
5		T24 052	T24 001	T24 003	T24 025	T24 014	
6		NR	NR	NR	NR	NR	
(bottom)							

Moderate and severe Alzheimer's disease

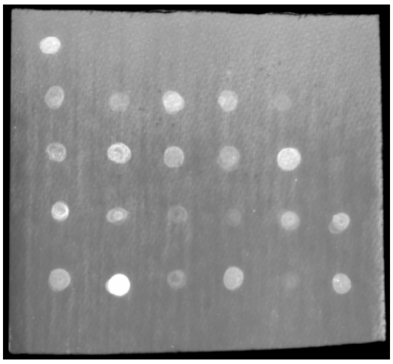

	(left)	1	2	3	4	5	6	(right)
(top)								
1		94-076						
2		DPM10/18	DPM11/09	DPM12/34	DPM13/30	DPM14/18		
3		DPM14/35	DPM15/46	DPM16/36	DPM16/37	DPM17/28		
4		DPM11/28	DPM12/01	DPM12/25	DPM13/10	DPM14/07	DPM14/10	
5		DPM14/30	DPM14/31	DPM14/50	DPM15/02	DPM16/10	DPM18/27	
(bottom)								

Supplementary Data s1 western and dot blot images

Parkinson's disorder

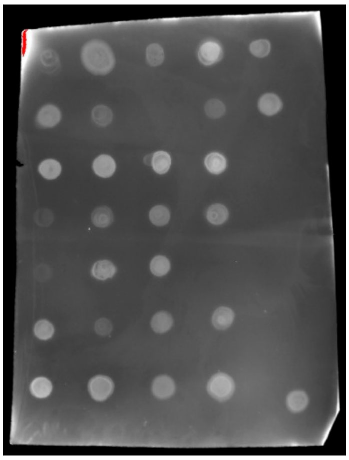

	(left)	1	2	3	4	5	(right)
**(top)**							
1		NR	NR	NR	NR	NR	
2		NR	NR	NR	NR	NR	
3		NR	NR	NR	NR		
4		NR	NR	NR	1994-076		
5		NR	NR	NR			
6		PD1001	PD1039	PD1040	PD1044		
7		PD1209	PD1217	PD1219	PD1221	NR	
(bottom)							

Non-Parkinson's

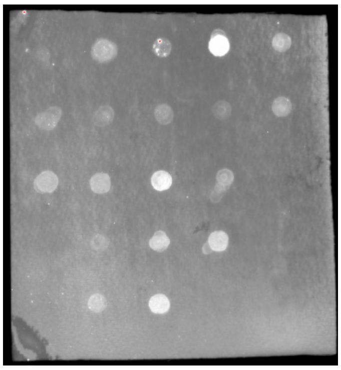

	(left)	1	2	3	4	5	(right)
**(top)**							
1		PDC030	PDC033	PDC035	PDC052	PDC059	
2		PDC067	PDC068	PDC069	PDC078	PDC084	
3		PDC114	PDC126	PDC128	PDC131		

Supplementary Data s1 western and dot blot images

4		NR	NR	NR	1994-076		
(bottom)		NR	NR	NR			
