# Supplementary material for: Accumulation of Cerebrospinal Fluid, Ventricular Enlargement, and Cerebral Folate Metabolic Errors Unify a Diverse Group of Neuropsychiatric Conditions Affecting Adult Neocortical Functions
Source: Int J Mol Sci. 2024 Sep 23;25(18):10205. doi: 10.3390/ijms251810205 (PMC11432090; doi:10.3390/ijms251810205)
Supplement: Supplementary file 1 [file ijms-25-10205-s001.zip › Supplementary TABLE S1 experimental data.pdf]

Supplementary TABLE s1 summarised data of all experiments

Table s1 showing summary of data collected from all samples including age and post-mortem delay (PMD) where available. FDH, FOLR1 and Folate show measurements normalised to the control of controls sample (1994-076) while Total protein is the actual measure of protein concentration. Outliers not included in mean calculations are highlighted in yellow and are those samples in which the average of 3 repeats is higher than 5x the median of its condition group with an average value higher than 0.2

	**Sample ID Brain bank number**	**Age** (y)	**PMD** (h)	**FDH**	**FOLR1**	**Folate**	**Total protein** (µg/µl)
Non-demented control	1994-076	78	8.4	1.000	1.000	1.000	4.090
	1992-026	83	6.4	0.955	0.799	0.134	4.430
	1992-030	78	6.6	2.420	1.879	0.908	4.331
	1990-019	70	20.1	5.836	3.105	2.159	6.711
	1990-059	84	4.4	0.571	0.588	0.256	2.292
	1990-031	82	5.3	2.309	2.988	1.859	6.841
	1993-012	92	12.5	8.564	7.047	4.171	5.123
Bipolar disorder	2000-088	73	5.3	0.079	0.355	0.100	1.815
	2015-069	64	8.1	0.170	0.448	0.786	3.352
	2006-075	80	9.5	1.614	1.282	0.872	2.831
	2000-111	70	4.8	0.443	1.077	0.438	2.619
	2012-127	68	4.9	6.976	3.402	0.706	4.396
	2014-070	66	7.6	12.002	1.465	1.542	6.518
	2007-076	79	7.4	0.170	1.599	0.605	1.873
	2014-041	79	8	4.085	1.479	1.193	4.632
	2013-038	72	4.6	0.168	2.147	0.770	3.069
	2015-077	72	9.9	3.069	0.265	0.629	4.695
	2015-031	51	4.5	0.890	0.893	2.460	3.264
	2015-044	83	15.4	2.495	3.559	1.064	2.943
	2012-048	81	6.7	0.070	0.258	0.304	1.481
Epilepsy	2002-045	59	7.1	6.697	1.806	2.593	6.488
	2008-081	91	6.8	0.016	1.591	0.188	1.628
	2010-087	76	3.8	5.453	2.672	2.622	3.885
	2015-059	86	8.1	0.006	0.581	0.895	3.477
	1995-074	77	16	14.160	3.382	2.009	4.883
	2015-093	86	11.8	1.528	0.251	2.328	3.941
Schizophrenia	2010-055	64	19.3	8.468	4.976	0.767	5.351
	2012-031	55	9.8	0.153	1.868	0.529	2.068
	1993-143	68	10.3	13.796	5.872	1.606	4.058
	2016-062	55	5.6	0.196	1.329	0.914	2.222
	2018-102	65	7.2	0.148	1.094	0.352	2.600
	2010-127	79	4.8	0.123	0.421	0.219	1.639
	2016-003	67	5.8	0.097	0.682	0.758	1.756
	1997-134	86	5.3	0.040	0.174	0.489	2.927
	2013-006	63	5	0.966	0.355	5.159	3.023

Supplementary TABLE s1 summarised data of all experiments

	2004-004	92	7.6	0.790	0.951	1.420	6.220
	2010-021	59	12.5	0.513	1.801	3.311	15.161
	2005-046	66	11.2	0.644	1.507	0.399	3.140
Multiple sclerosis	MS023	74	18	1.795	0.341	0.729	4.568
	MS061	56	6	1.192	0.467	0.329	2.117
	MS026	78	8	0.476	1.276	0.286	2.488
	MS071	78	5	0.173	0.042	1.075	1.108
	MS387	42	13	1.660	1.181	0.869	2.331
	MS125	76	13	1.002	1.546	0.352	2.508
	MS543	67	11	3.763	2.095	0.335	2.488
	MS115	75	21	2.368	1.050	0.264	4.892
	MS547	66	17	3.090	1.616	0.223	3.733
	MS086	81	15	0.582	2.094	0.092	1.604
	MS049	75	8	6.493	0.497	0.470	2.655
	MS199	87	15	0.354	0.380	1.245	4.868
	MS407	44	22	1.004	0.132	0.583	2.106
	MS411	61	24	3.922	0.563	0.235	0.788
	MS528	45	17	2.316	0.340	1.004	2.223
Live / dementia	LW1	68	-	0.001	0.011	0.035	0.648
	LW2	51	-	0.002	0.014	0.083	0.889
	LW3	58	-	0.008	0.001	0.000	0.912
	LW4	67	-	0.005	0.005	0.010	1.049
	LW5	61	-	0.005	0.007	0.036	0.590
	LW6	63	-	0.052	0.005	0.015	0.614
	LW7	67	-	0.035	0.019	0.172	0.930
	LW8	54	-	0.011	0.002	0.002	0.660
	LW9	-	-	0.020	0.000	0.023	1.019
Brain injury	HI 11/28	71	64	0.767	0.386	1.476	2.724
	HI 14/34	94	74.5	1.214	0.055	5.139	10.106
	HI 16/11	77	63	2.035	0.159	2.167	5.283
	HI 16/31	90	155	3.571	0.228	4.280	10.805
	HI 17/06	75	82	1.779	1.446	0.647	5.639
	HI 17/23	91	88	0.743	0.319	1.657	3.649
	HI 18/27	75	104	3.079	0.376	1.765	4.132
	HI 18/35	85	85	1.599	0.369	2.945	2.670
	HI 18/39	75	127	1.663	0.586	2.488	4.172
Idiopathic intracranial hypertension	IIH093	-	-	0.039	0.005	0.080	0.515
	IIH006	-	-	0.021	0.000	0.102	1.165
	IIH028	-	-	0.019	0.003	0.058	0.588
	IIH058	-	-	0.014	0.000	0.054	0.688
	IIH209	-	-	0.006	0.009	0.081	1.008
	IIH062	-	-	0.020	0.038	0.090	0.547
	IIH007	-	-	0.033	0.061	0.061	0.525
	IIH009	-	-	0.009	0.011	0.027	0.751

Supplementary TABLE s1 summarised data of all experiments

	IIH036	-	-	0.007	0.001	0.084	0.818
	IIH208	-	-	0.007	0.001	0.081	0.800
Normal pressure hydrocephalus T0	T0 040	-	-	0.040	0.004	0.183	0.285
	T0 058	-	-	0.005	0.004	0.053	2.037
	T0 013	-	-	0.002	0.001	0.178	1.118
	T0 044	-	-	0.012	0.002	0.047	0.660
	T0 052	-	-	0.009	0.002	0.028	0.824
	T0 004	68	-	0.023	0.012	0.124	0.655
	T0 057	-	-	0.057	0.010	0.331	1.007
	T0 041	79	-	0.020	0.000	0.383	1.106
	T0 001	77	-	0.010	0.000	0.015	2.429
	T0 003	66	-	0.008	0.000	0.373	0.792
	T0 025	62	-	0.033	0.003	0.282	1.040
	T0 033	77	-	0.000	0.000	0.083	0.915
	T0 045	73	-	0.011	0.000	0.123	0.670
	T0 049	57	-	0.000	0.000	0.225	0.848
	T0 056	85	-	0.012	0.000	0.023	0.660
	T0 014	-	-	0.000	0.000	0.528	1.094
Normal pressure hydrocephalus T24	T24 052	As above for T0		0.003	0.002	0.907	1.091
	T24 041			0.000	0.002	0.121	1.293
	T24 004			0.001	0.002	0.067	0.623
	T24 057			0.002	0.005	0.173	1.123
	T24 044			0.001	0.012	0.639	1.190
	T24 001			0.012	0.000	0.659	1.192
	T24 003			0.003	0.000	0.464	0.806
	T24 025			0.013	0.002	1.144	1.004
	T24 033			0.013	0.000	0.038	1.046
	T24 049			0.000	0.003	0.086	0.735
	T24 045			0.043	0.003	0.064	1.048
	T24 056			0.018	0.000	0.219	1.542
	T24 014			0.037	0.004	3.078	3.441
Moderate Alzheimer's disease	DPM10/18	97	25	0.681	0.013	0.561	2.372
	DPM11/09	91	33	0.793	0.040	0.337	1.956
	DPM12/34	80	81	2.231	1.020	1.060	5.198
	DPM13/30	77	87	0.264	0.708	0.398	3.835
	DPM14/18	77	162	5.040	0.356	0.196	4.113
	DPM14/35	83	56	1.468	0.282	0.600	1.301
	DPM15/46	76	60.5	0.150	0.252	1.026	3.714
	DPM16/36	75	71.5	1.465	0.787	0.625	6.843
	DPM16/37	86	93.5	2.320	4.307	0.573	4.903
	DPM17/28	80	55.5	1.927	5.405	1.076	5.978
Severe Alzheimer's disease	DPM11/28	71	64	0.038	0.368	0.871	1.716
	DPM12/01	67	84	0.108	0.019	0.621	4.319
	DPM12/25	62	50.5	0.127	0.323	0.170	3.551

Supplementary TABLE s1 summarised data of all experiments

	DPM13/10	85	24	0.100	0.544	0.153	2.123
	DPM14/07	78	39	0.590	0.022	0.421	3.910
	DPM14/10	78	70	1.150	0.438	0.711	2.895
	DPM14/30	70	89	1.602	2.361	0.584	7.987
	DPM14/31	64	98.5	2.521	0.848	2.207	7.500
	DPM14/50	63	54	0.149	0.000	0.261	2.261
	DPM15/02	78	173	0.865	1.768	0.928	6.625
	DPM16/10	59	87	0.038	0.068	0.154	1.587
	DPM18/27	75	104	1.284	0.729	0.783	5.926
Parkinson's disease	PD1001	88	33	1.570	0.554	0.979	6.130
	PD1039	87	46	0.613	1.033	0.366	3.563
	PD1040	82	33			0.761	5.545
	PD1044	82	11	0.616	0.194	0.970	4.055
	PD1209	71	-	5.601	0.179	1.220	8.886
	PD1216	84	-	0.610	0.516		7.882
	PD1217	74	-	2.965	0.366	1.271	12.204
	PD1219	77	-	4.671	0.494	0.294	8.332
	PD1221	78	-	0.973	0.125	0.262	5.546
Parkinson's disease Control	PDC030	87	17	2.071	0.299	0.270	5.354
	PDC033	77	43	5.426	0.704	1.871	11.110
	PDC035	89	13	1.686	1.724	0.657	4.670
	PDC052	73	30	1.227	0.430	2.328	5.131
	PDC059	82	15	0.948	0.082	0.900	3.423
	PDC067	92	35	3.556	1.616	0.929	5.864
	PDC068	95	28	0.499	0.299	0.433	7.097
	PDC069	90	27	2.398	0.125	1.114	3.759
	PDC078	91	18	1.819	0.425	0.439	2.561
	PDC084	83	26	4.441	0.039	1.142	4.848
	PDC114	70	36	1.533	1.114	1.410	6.986
	PDC126	82	20	2.400	0.619	1.605	8.359
	PDC128	91	22	1.373	0.235	1.619	3.353
	PDC131	92	24	1.406	0.193	1.572	2.955
