# Supplementary material for: Accumulation of Cerebrospinal Fluid, Ventricular Enlargement, and Cerebral Folate Metabolic Errors Unify a Diverse Group of Neuropsychiatric Conditions Affecting Adult Neocortical Functions
Source: Int J Mol Sci. 2024 Sep 23;25(18):10205. doi: 10.3390/ijms251810205 (PMC11432090; doi:10.3390/ijms251810205)
Supplement: Supplementary file 1 [file ijms-25-10205-s001.zip › Supplementary TABLE S2 experimental data.pdf]

Supplementary TABLE S2 summarised data of all experiments

Table showing summary of data collected from all samples. FDH, FOLR1 and Folate show measurements normalised to the control of controls sample (1994-076) while Total protein is the actual measure of protein concentration. Outliers not included in mean calculations are highlighted in yellow and are those samples in which the average of 3 repeats is higher than 5x the median of its condition group with an average value higher than 0.2

	Sample ID	**FDH**	**FOLR1**	**Folate**	**Total protein (ug/ul)**
Non-demented control	1994-076	1.000	1.000	1.000	4.090
	1992-026	0.955	0.799	0.134	4.430
	1992-030	2.420	1.879	0.908	4.331
	1990-019	5.836	3.105	2.159	6.711
	1990-059	0.571	0.588	0.256	2.292
	1990-031	2.309	2.988	1.859	6.841
	1993-012	8.564	7.047	4.171	5.123
Bipolar disorder	2000-088	0.079	0.355	0.100	1.815
	2015-069	0.170	0.448	0.786	3.352
	2006-075	1.614	1.282	0.872	2.831
	2000-111	0.443	1.077	0.438	2.619
	2012-127	6.976	3.402	0.706	4.396
	2014-070	12.002	1.465	1.542	6.518
	2007-076	0.170	1.599	0.605	1.873
	2014-041	4.085	1.479	1.193	4.632
	2013-038	0.168	2.147	0.770	3.069
	2015-077	3.069	0.265	0.629	4.695
	2015-031	0.890	0.893	2.460	3.264
	2015-044	2.495	3.559	1.064	2.943
	2012-048	0.070	0.258	0.304	1.481
Epilepsy	2002-045	6.697	1.806	2.593	6.488
	2008-081	0.016	1.591	0.188	1.628
	2010-087	5.453	2.672	2.622	3.885
	2015-059	0.006	0.581	0.895	3.477
	1995-074	14.160	3.382	2.009	4.883
	2015-093	1.528	0.251	2.328	3.941
Schizophrenia	2010-055	8.468	4.976	0.767	5.351
	2012-031	0.153	1.868	0.529	2.068
	1993-143	13.796	5.872	1.606	4.058
	2016-062	0.196	1.329	0.914	2.222
	2018-102	0.148	1.094	0.352	2.600
	2010-127	0.123	0.421	0.219	1.639
	2016-003	0.097	0.682	0.758	1.756
	1997-134	0.040	0.174	0.489	2.927
	2013-006	0.966	0.355	5.159	3.023

Supplementary TABLE S2 summarised data of all experiments

	2004-004	0.790	0.951	1.420	6.220
	2010-021	0.513	1.801	3.311	15.161
	2005-046	0.644	1.507	0.399	3.140
Multiple sclerosis	MS023	1.795	0.341	0.729	4.568
	MS061	1.192	0.467	0.329	2.117
	MS026	0.476	1.276	0.286	2.488
	MS071	0.173	0.042	1.075	1.108
	MS387	1.660	1.181	0.869	2.331
	MS125	1.002	1.546	0.352	2.508
	MS543	3.763	2.095	0.335	2.488
	MS115	2.368	1.050	0.264	4.892
	MS547	3.090	1.616	0.223	3.733
	MS086	0.582	2.094	0.092	1.604
	MS49	6.493	0.497	0.470	2.655
	MS199	0.354	0.380	1.245	4.868
	MS407	1.004	0.132	0.583	2.106
	MS411	3.922	0.563	0.235	0.788
	MS528	2.316	0.340	1.004	2.223
Live / dementia	LW1	0.001	0.011	0.035	0.648
	LW2	0.002	0.014	0.083	0.889
	LW3	0.008	0.001	0.000	0.912
	LW4	0.005	0.005	0.010	1.049
	LW5	0.005	0.007	0.036	0.590
	LW6	0.052	0.005	0.015	0.614
	LW7	0.035	0.019	0.172	0.930
	LW8	0.011	0.002	0.002	0.660
	LW9	0.020	0.000	0.023	1.019
Brain injury	HI 11/28	0.767	0.386	1.476	2.724
	HI 14/34	1.214	0.055	5.139	10.106
	HI 16/11	2.035	0.159	2.167	5.283
	HI 16/31	3.571	0.228	4.280	10.805
	HI 17/06	1.779	1.446	0.647	5.639
	HI 17/23	0.743	0.319	1.657	3.649
	HI 18/29	3.079	0.376	1.765	4.132
	HI 18/35	1.599	0.369	2.945	2.670
	HI 18/39	1.663	0.586	2.488	4.172
Idiopathic intracranial hypertension	IIH093	0.039	0.005	0.080	0.515
	IIH006	0.021	0.000	0.102	1.165
	IIH028	0.019	0.003	0.058	0.588
	IIH058	0.014	0.000	0.054	0.688
	IIH209	0.006	0.009	0.081	1.008
	IIH062	0.020	0.038	0.090	0.547
	IIH007	0.033	0.061	0.061	0.525
	IIH009	0.009	0.011	0.027	0.751

Supplementary TABLE S2 summarised data of all experiments

	IIH036	0.007	0.001	0.084	0.818
	IIH208	0.007	0.001	0.081	0.800
Normal pressure hydrocephalus T0	T0 040	0.040	0.004	0.183	0.285
	T0 058	0.005	0.004	0.053	2.037
	T0 013	0.002	0.001	0.178	1.118
	T0 044	0.012	0.002	0.047	0.660
	T0 052	0.009	0.002	0.028	0.824
	T0 004	0.023	0.012	0.124	0.655
	T0 057	0.057	0.010	0.331	1.007
	T0 041	0.020	0.000	0.383	1.106
	T0 001	0.010	0.000	0.015	2.429
	T0 003	0.008	0.000	0.373	0.792
	T0 025	0.033	0.003	0.282	1.040
	T0 033	0.000	0.000	0.083	0.915
	T0 045	0.011	0.000	0.123	0.670
	T0 049	0.000	0.000	0.225	0.848
	T0 056	0.012	0.000	0.023	0.660
	T0 014	0.000	0.000	0.528	1.094
Normal pressure hydrocephalus T24	T24 052	0.003	0.002	0.907	1.091
	T24 041	0.000	0.002	0.121	1.293
	T24 004	0.001	0.002	0.067	0.623
	T24 057	0.002	0.005	0.173	1.123
	T24 044	0.001	0.012	0.639	1.190
	T24 001	0.012	0.000	0.659	1.192
	T24 003	0.003	0.000	0.464	0.806
	T24 025	0.013	0.002	1.144	1.004
	T24 033	0.013	0.000	0.038	1.046
	T24 049	0.000	0.003	0.086	0.735
	T24 045	0.043	0.003	0.064	1.048
	T24 056	0.018	0.000	0.219	1.542
	T24 014	0.037	0.004	3.078	3.441
Moderate Alzheimer's disease	DPM10/18	0.681	0.013	0.561	2.372
	DPM11/09	0.793	0.040	0.337	1.956
	DPM12/34	2.231	1.020	1.060	5.198
	DPM13/30	0.264	0.708	0.398	3.835
	DPM14/18	5.040	0.356	0.196	4.113
	DPM14/35	1.468	0.282	0.600	1.301
	DPM15/46	0.150	0.252	1.026	3.714
	DPM16/36	1.465	0.787	0.625	6.843
	DPM16/37	2.320	4.307	0.573	4.903
	DPM17/28	1.927	5.405	1.076	5.978
Severe Alzheimer's disease	DPM11/28	0.038	0.368	0.871	1.716
	DPM12/01	0.108	0.019	0.621	4.319
	DPM12/25	0.127	0.323	0.170	3.551

Supplementary TABLE S2 summarised data of all experiments

	DPM13/10	0.100	0.544	0.153	2.123
	DPM14/07	0.590	0.022	0.421	3.910
	DPM14/10	1.150	0.438	0.711	2.895
	DPM14/30	1.602	2.361	0.584	7.987
	DPM14/31	2.521	0.848	2.207	7.500
	DPM14/50	0.149	0.000	0.261	2.261
	DPM15/02	0.865	1.768	0.928	6.625
	DPM16/10	0.038	0.068	0.154	1.587
	DPM18/27	1.284	0.729	0.783	5.926
Parkinson's disease	PD1001	1.570	0.554	0.979	6.130
	PD1039	0.613	1.033	0.366	3.563
	PD1040			0.761	5.545
	PD1044	0.616	0.194	0.970	4.055
	PD1209	5.601	0.179	1.220	8.886
	PD1216	0.610	0.516		7.882
	PD1217	2.965	0.366	1.271	12.204
	PD1219	4.671	0.494	0.294	8.332
	PD1221	0.973	0.125	0.262	5.546
Parkinson's disease Control	PDC030	2.071	0.299	0.270	5.354
	PDC033	5.426	0.704	1.871	11.110
	PDC035	1.686	1.724	0.657	4.670
	PDC052	1.227	0.430	2.328	5.131
	PDC059	0.948	0.082	0.900	3.423
	PDC067	3.556	1.616	0.929	5.864
	PDC068	0.499	0.299	0.433	7.097
	PDC069	2.398	0.125	1.114	3.759
	PDC078	1.819	0.425	0.439	2.561
	PDC084	4.441	0.039	1.142	4.848
	PDC114	1.533	1.114	1.410	6.986
	PDC126	2.400	0.619	1.605	8.359
	PDC128	1.373	0.235	1.619	3.353
	PDC131	1.406	0.193	1.572	2.955
